# Postconditioning improvement effects of ulinastatin on brain injury following cardiopulmonary resuscitation

**DOI:** 10.3892/etm.2014.1876

**Published:** 2014-08-04

**Authors:** BO SUI, YONGWANG LI, LI MA

**Affiliations:** 1 Department of Anesthesiology, The Second Artillery General Hospital, Beijing 100088, P.R. China; 2 Department of Gynecology and Obstetrics, The Second Artillery General Hospital, Beijing 100088, P.R. China

**Keywords:** cardiac arrest, cardiopulmonary resuscitation, ulinastatin, inflammation, Toll-like receptor 4 signaling pathway

## Abstract

The aim of the present study was to determine the effects of ulinastatin (UTI) on brain injury in rats subjected to cardiopulmonary resuscitation (CPR) following asphyxial cardiac arrest (CA) and identify the underlying mechanisms. In total, 100 healthy male Wistar rats were randomly divided into control and treatment groups (n=50). After 4 min of asphyxial CA, all the rats were immediately subjected to CPR. The treatment group animals were administered 15 mg/kg UTI at the onset of resuscitation. The mortality rate in the two groups was recorded at 24 h post-resuscitation. In addition, neurological function was evaluated at 24, 48 and 72 h post-resuscitation using a neurological deficit scale (NDS). Furthermore, the effects of UTI on the Toll-like receptor 4 (TLR4) signaling pathway in brain tissues were determined by assessing TLR4 mRNA expression, nuclear factor (NF)-κB activity and tumor necrosis factor (TNF)-α and interleukin (IL)-6 levels at 1, 3, 6, 12, 24, 48 and 72 h post-resuscitation. After 24 h, the mortality rate significantly decreased in the treatment group when compared with the control animals (10 vs. 30%; P<0.05). Additionally, an overt improvement was observed in the NDS score following UTI treatment when compared with the control (P<0.01). Finally, statistically significant decreases in the levels of TLR4 mRNA expression, NF-κB activity and TNF-α and IL-6 were observed in the treatment group at each time point (P<0.01). Therefore, UTI treatment at the onset of CPR significantly inhibits the TLR4 signaling pathway, thereby alleviating the inflammatory responses following resuscitation and improving neurological function.

## Introduction

Statistical data show that ~1 million patients with cardiac arrest (CA) are recorded annually in the United States and Europe (1). However, treatment of CA with cardiopulmonary resuscitation (CPR) often leads to an unsatisfactory outcome. For example, 30–40% of patients that have undergone CPR achieve a return of spontaneous circulation (ROSC), 10–30% are completely healed and discharged from hospital, while the remaining patients eventually succumb to the illness. In addition, patients that have been successfully resuscitated can often experience permanent neurological complications, including cognitive impairment and motor deficit (2,3). Therefore, providing an effective regimen for the treatment and prevention of neurological injury following CPR is important.

Organ injury following successful CPR from CA is considered to be associated with systemic inflammatory responses; levels of various cytokines and lipopolysaccharide (LPS) have been shown to be markedly elevated in patients that have been successfully resuscitated from CA (4,5). LPS can be identified by the cell-surface Toll-like receptor 4 (TLR4), which induces inflammatory responses and promotes the production of a number of inflammatory cytokines, resulting in a sepsis-like syndrome. The dynamic changes in the levels of inflammatory cytokines and LPS have been shown to be closely associated with prognosis in patients that have undergone CPR (6). A previous study demonstrated that neurological injury following CPR is associated with the activation of cerebral inflammatory responses, with the TLR4 signaling pathway playing a contributory role in the occurrence of neurological injury (7). Under cerebral ischemia and anoxia, the activated TLR4 promotes the expression of nuclear factor (NF)-κB and induces the production of cytokines, such as tumor necrosis factor (TNF)-α and interleukin (IL)-6, thereby causing inflammation and neurological injury (7). Therefore, it is hypothesized that the TLR4 signaling pathway may be inhibited by hypoxic preconditioning to attenuate inflammatory responses and neurological injury; thus, protecting brain function. Notably, a significantly reduced area of cerebral infarction and an alleviation of inflammatory responses in the brain have been observed in TLR4 knockout mice following cerebral ischemia (8). However, attenuation of brain injury following CA by hypoxic preconditioning remains difficult in clinical settings. Therefore, postconditioning with an effective medicine is of vital importance.

Ulinastatin (UTI), a urinary trypsin inhibitor extracted and purified from human urine, has been shown to possess anti-inflammatory properties, suppressing the infiltration of neutrophils and the release of elastinase and chemokines (9). In addition, UTI protects mitochondrial function by reducing the calcium overload in injured cells and exhibits protective effects against ischemia-reperfusion (I/R) injury in the heart, lung, liver and kidney (9). Furthermore, previous studies have demonstrated that UTI can alleviate LPS-induced lung and kidney injury by inhibiting inflammatory responses in these organs (10,11). Thus, we hypothesized that UTI may protect against cerebral I/R injury following CPR and inhibit TLR4-induced inflammatory responses in the brain following resuscitation. In the present study, a rat model of CPR following asphyxial CA was established, in which the effects of UTI on the TLR4 signaling pathway were evaluated, as well as the protective mechanisms against cerebral I/R injury in rats subjected to CPR following CA.

The effects of UTI were investigated with regard to the mortality rate, TLR4 mRNA expression, NF-κB activity and the levels of TNF-α and IL-6. The aim of the present study was to determine whether UTI improved neurological function; thus, may be used in combination with CPR for the treatment of brain injury following CA.

## Materials and methods

### Animal treatments

**Figure 1:**
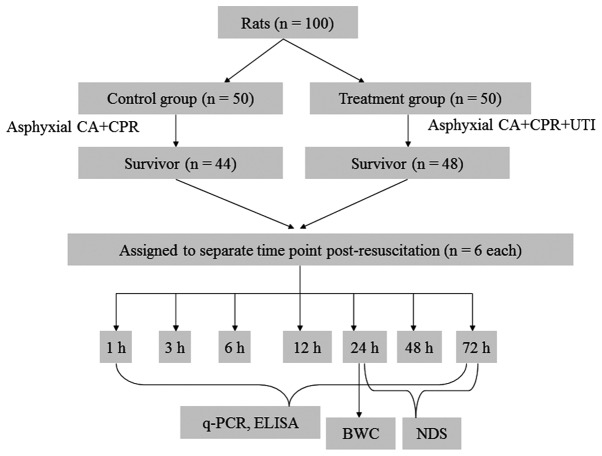
Disposition of rats in the two groups. CA, cardiac arrest; CPR, cardiopulmonary resuscitation; UTI, ulinastatin; qPCR, quantitative polymerase chain reaction; ELISA, enzyme-linked immunosorbent assay; BWC, brain water content; NDS, neurological deficit scale.

The study was approved by the Experimental Animal Protection and Ethics Committee of the Second Artillery General Hospital (Beijing, China). In total, 100 male Wistar rats (age, 2 months; weight, 250–350 g) were randomly assigned to control and treatment groups (n=50). After 4 min of asphyxial CA, all the rats were immediately subjected to CPR. The treatment group animals were administered 15 mg/kg UTI (Techpool Bio-Pharma, Guangdong, China) at the onset of resuscitation (Fig. 1).

### Establishment of a rat model of CPR following asphyxial CA

Asphyxial CA was induced as previously described (12–16). Rats in the two groups were anesthetized with 30 mg/kg chloral hydrate, orally intubated and mechanically ventilated using a small-animal ventilator. The tidal volume, respiratory rate, inspiratory/expiratory ratio (I/E) and the fraction of inspired oxygen were set at 8 ml/kg, 40 breaths/min, 1/2 and 1.0, respectively. Next, the rats were intravenously injected with 2 mg/kg vecuronium bromide to prevent spontaneous respiration. The rectal temperature was maintained at 37.5±0.3°C with a heating lamp during the induction of CA. The femoral vein and artery were cannulated in order to provide fluid and medications and to monitor the arterial pressure, heart rate (HR) and arterial blood gas.

Baseline physiological variables were recorded continuously for 10 min following surgery. CA was defined as a reduction in the mean arterial pressure (MAP) to a value of <10 mmHg after ventilation had been discontinued for ~3 min. After 1 min of CA, CPR was performed with continuous ventilation and external cardiac compressions at a rate of 200/min, along with an intravenous injection of 0.01 mg/kg epinephrine and 1 mmol/kg sodium bicarbonate. Successful CPR was defined as an achievement of ROSC, indicated by a rise in the MAP to a value of >50 mmHg. CPR was considered to have failed if no sign of ROSC was observed during the 5 min post-resuscitation.

Following resuscitation, the rats were mechanically ventilated with an increased I/E ratio of 1/1.5 and treated with a continuous infusion of 8 ml/h lactated Ringer’s solution until the restoration of adequate spontaneous respiration. The rats were then weaned from the ventilator, extubated and observed for 10 min. This was followed by arteriovenous ligation and skin sutures if there were no abnormalities observed in the circulation and respiration. Following restoration in an oxygen tank for 30 min, the rats were finally returned to their respective cages with access to food and water *ad libitum*.

### Determination of the mortality rate

The mortality rate in the two groups was recorded at 24 h post-resuscitation.

### Assessment of the circulatory function

MAP and HR were determined for all the animals during the first 60 min post-resuscitation. The time to CA (TCA) and time to ROSC (TROSC) were also recorded. The TCA was defined as the time from the initiation of ventilation to the onset of CA, while the TROSC was defined as the time from the start of resuscitation to ROSC.

### Sample collection

At 1, 3, 6, 12, 24, 48 and 72 h post-resuscitation, all the rats were decapitated, and their brains quickly removed and placed on ice for dissection. Following washing in ice-cold normal saline, the surface water on the organs was gently blotted off with filter paper. Left hemisphere brain tissues were stored in liquid nitrogen, until required for the analysis of TLR4 mRNA expression and NF-κB, TNF-α and IL-6 protein levels.

### Determination of brain water content (BWC)

A small sample of brain tissue (~50 mg) from the right hemisphere was extracted at 24 h post-resuscitation, as described previously. The brain tissues were immediately weighed on an electronic analytical balance, dried at 105°C for 24 h and weighed again. The BWC was calculated as follows: (wet weight - dry weight)/wet weight × 100%.

### Neurological assessment

Neurological function was evaluated in the rats at 24, 48 and 72 h post-resuscitation using a neurological deficit scale (NDS) scoring system (score range, 0–100; score 0, normal; score 100, brain dead; Table I) (13,14).

### Isolation of total RNA and cDNA synthesis

Total RNA was extracted from the brain tissue specimens of rats using a TriPure isolation reagent (Roche Diagnostics, Mannheim, Germany), according to the manufacturer’s instructions. Briefly, fresh or frozen tissues (~100 mg) were homogenized in 1 ml TriPure reagent and submitted to centrifugation at 21,578 × g for 10 min at 4°C. Following the addition of 0.2 ml chloroform to the supernatants, the mixture was incubated at room temperature for 20 min with occasional stirring. The samples were then submitted to centrifugation again, as aforementioned. The resulting supernatants were mixed with 0.5 ml isopropanol, incubated for 5–10 min and centrifuged as aforementioned. The pellet containing total RNA was washed with 1 ml ethanol (75%) and centrifuged at 8,429 × g for 5 min at 4°C. The RNA pellet was air dried, dissolved in 3 ml diethylpyrocarbonate-treated water and the optical density (OD) values were measured at 260 and 280 nm on a Lambda 20 UV/Vis spectrophotometer (Perkin-Elmer, Norwalk, CT, USA). The RNA concentration was calculated as follows: RNA concentration (μg/ml) = OD_260_ × dilution factor × 40/1,000. RNA purity was assessed by the OD_260_/OD_280_ ratio.

### Detection of TLR4 mRNA expression levels

TLR4 gene expression was evaluated using quantitative polymerase chain reaction (qPCR). Plasmids of TLR4 and the housekeeping gene, glyceraldehyde-3-phosphate dehydrogenase (GAPDH), were obtained at 3.67 and 2.67×10^10^ copies/μl, respectively, and diluted with sterile water to final concentrations of 1×10^6^, 1×10^5^, 1×10^4^ and 1×10^3^ copies/μl. The qPCR mixture (25 μl) contained 1X PCR buffer, 4 mM MgCl_2_, 0.4 μM each primer, 0.4 μM *Taq*Man probe, 0.5 units AmpErase Uracil N-Glycosylase, 0.5 units Ampli*Taq* Gold DNA polymerase, 50 ng cDNA, double distilled water, 0.2 mM dUTP and 0.1 mM dATP, dGTP and dCTP.

qPCR was conducted using an ABI Prism 7500 SDS RT-PCR system (Applied Biosystems, Foster City, CA, USA). The reaction conditions for the amplification of TLR4 and GAPDH were 40 cycles of 50°C for 2 min, 95°C for 2 min, 95°C for 15 sec and 60°C for 60 sec. The amplified products for TLR4 and GAPDH were 122 and 138 bp in size, respectively. The primers and fluorescent probes used for qPCR were as follows: TLR4 forward, 5′-TGAGAAACGAGCTGGTAAAGAATT-3′, reverse, 5′-GTGGAAGCCTTCCTGGATGATG-3′; and probe, 5′-AGTGCCCCGCTTTCAGCTTTGCCT-3′; GAPDH forward, 5′-CATGACCTTCCGTGTTCCTACC-3′, reverse, 5′-TAGCCCAGGATGCCCTTCAG-3′ and probe, 5′-CCTCAGACGCCTGCTTCACCACCT-3′.

The threshold level was set at 10-times the standard deviation (SD) of the baseline fluorescence measured during PCR cycles 3–15. The threshold cycle (Ct) was defined as the cycle number at which fluorescence passed the fixed threshold, normalized against GAPDH. Standard curves of the Ct values (serial dilutions of the control plasmids plotted against the logarithm of the copy numbers) were constructed for TLR4 and GAPDH. The correlation coefficients of the standard curves for TLR4 and GAPDH were 0.986 and 0.971, respectively. The absolute copy numbers of TLR4 mRNA in each unknown sample were then calculated based on the Ct values. The GAPDH mRNA copy number in each sample was used to normalize TLR4 mRNA expression levels.

### Extraction of nucleoproteins from the brain tissues

Nucleoproteins were extracted from the brain tissues using a nucleoprotein extraction kit (Active Motif, Carlsbad, CA, USA). Briefly, ~200 mg tissue samples were homogenized in 2 ml nuclear extraction buffer (NEB) A. The homogenates were then centrifuged at 599 × g for 30 sec, and the supernatants containing the complete nuclei were incubated on ice for 5 min. Following centrifugation at 3,746 × g for 10 min at 4°C, the nuclear precipitates (pellet) were resuspended in 150 μl NEB B and incubated for 30 min on ice. A final centrifugation at 29,371 × g for 1 min at 4°C yielded nucleoproteins in the supernatants, which were stored at −70°C until required for the assessment of protein concentrations using the Coomassie Brilliant Blue G-250 method (8).

### Detection of NF-κB activity

NF-κB activity levels in the nuclear extracts were detected by a specific high-sensitive enzyme-linked immunosorbent assay (ELISA), using a TransAM NF-κB detection kit (Active Motif), according to the manufacturer’s instructions. Antibodies were used at a 1:1,000 dilution in antibody binding buffer and incubations were performed at room temperature for 1 h. The reactions were visualized using 100 μl tetramethylbenzidine (TMB) chromogenic substrate for 10 min in the dark, and were stopped following the addition of 100 μl stop solution. Absorbance was measured immediately at 450 nm on a microplate reader.

### Detection of TNF-α and IL-6 levels

TNF-α and IL-6 levels were assessed using specific ELISA kits (Sigma-Aldrich, St. Louis, MO, USA). Briefly, samples or standards (2,000, 1,000, 500, 250 and 125 pg/ml) were added to the wells. Following incubation for 1 h at 37°C, and four washes in 350 μl washing solution, the plates were incubated in the presence of 100 μl biotin antibodies for 1 h at 37°C. Next, the plates were washed and incubated with 100 μl enzyme binding buffer for 30 min at 37°C. The reactions were visualized using 100 μl TMB chromogenic substrate for 15–20 min in the dark, and stopped following the addition of 100 μl stop solution. Absorbance was measured within 15 min at 450 nm on a microplate reader. Based on the measured OD values, the protein levels of TNF-α and IL-6 were determined using the corresponding standard curves.

### Statistical analysis

Statistical analyses were performed using SPSS statistical software version 12.0 (SPSS, Inc., Chicago, IL, USA). All the data are expressed as the mean ± SD. The Student’s t-test was used to compare differences between the two groups, where P<0.05 was considered to indicate a statistically significant difference, and P<0.01 indicated a higher degree of statistical significance.

## Results

### Baseline characteristics

No statistically significant differences were observed in the baseline characteristics, including the body weight, HR, MAP and NDS, between the two groups (data not shown).

### Analysis of the mortality rate, TCA, TROSC and BWC

As shown in Table II, at 24 h post-resuscitation, six and two mortalities were recorded in the control and treatment groups, respectively; a statistically significant difference was observed in the mortality rates (P<0.01). TROSC values were significantly higher in the control animals as compared with the treatment group (99.3±16.6 vs. 73.9±22.4 sec; P<0.01). In addition, the BWC was markedly reduced following treatment with UTI when compared with the control rats (77.33±4.55 vs. 84.06±1.83; P<0.01). However, no statistically significant difference in the TCA was observed between the control and treatment groups (191.1±20.8 vs. 190.3±19.6 sec; P>0.05).

### Evaluation of neurological function using the NDS scores

Neurological data are summarized in Table II. The NDS scores were similar at the baseline in the two groups of rats. At 24, 48 and 72 h post-resuscitation, the NDS scores were significantly higher in the treatment group when compared with control animals (P<0.01). In addition, a statistically significant increase was observed in the NDS scores at 48 h post-resuscitation when compared with the values obtained at 24 h for each group (P<0.05); the trend continued to 72 h.

### Effect of UTI on TLR4 gene expression levels

**Figure 2:**
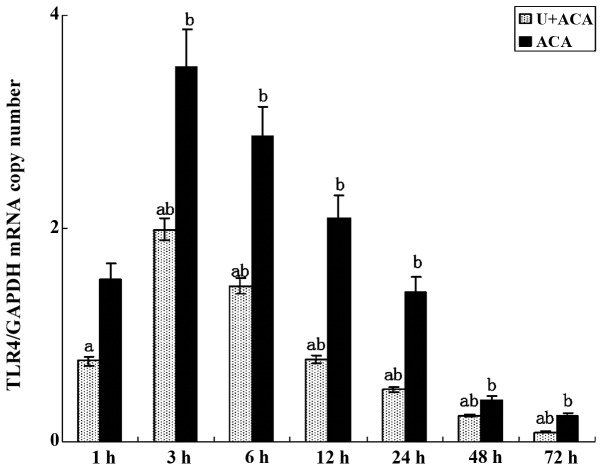
Effect of UTI on TLR4 gene expression in the control and treatment groups (n=6). ^a^P<0.01, vs. ACA group; ^b^P<0.01, vs. previous time point. TLR4, Toll-like receptor 4; U or UTI/U, ulinastatin; ACA, asphyxial cardiac arrest.

According to the qPCR results (Fig. 2), TLR4 mRNA expression was detected in the brain tissues collected from all the animals at 1 h after resuscitation. Peak expression was observed between 3 and 6 h, prior to markedly decreasing after 12 h, although expression remained detectable at 72 h. Notably, TLR4 gene expression was significantly reduced in the treatment group as compared with the control rats at each time point (P<0.05).

### Evaluation of NF-κB activity levels

**Figure 3:**
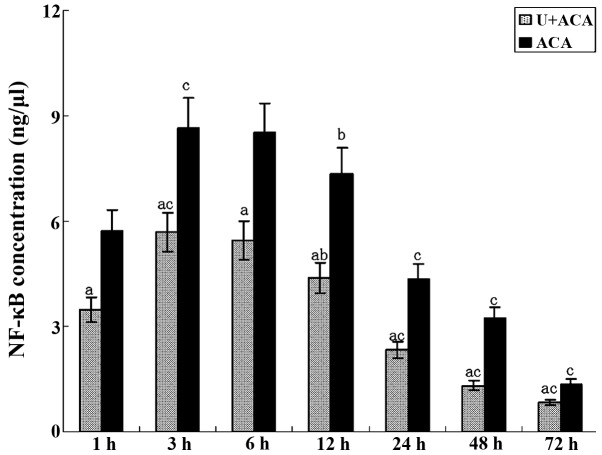
Effect of UTI on NF-κB activity. ^a^P<0.01, vs. ACA group; ^b^P<0.05 and ^c^P<0.01, vs. previous time point (n=6). U or UTI, ulinastatin; NF, nuclear factor; ACA, asphyxial cardiac arrest.

As shown in Fig. 3, NF-κB activity was detected at 1 h post-resuscitation in the two groups. The values increased and peaked between 3 and 6 h. At 24 h, NF-κB activity was significantly reduced and continued to decrease further, although a degree of activity was detected at 72 h. Notably, a significant reduction in the NF-κB activity levels was observed in the treatment group at each time point when compared with the control animals (P<0.05). NF-κB activity levels were consistent with the trends observed for TLR4 mRNA expression.

### Effect of UTI on TNF-α and IL-6 production

**Figure 4:**
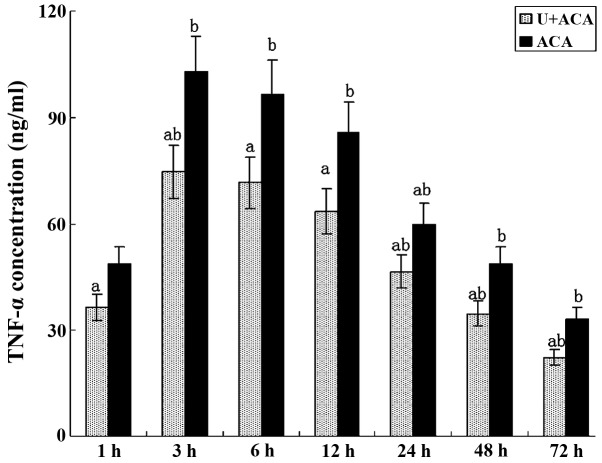
Effect of UTI on TNF-α levels. ^a^P<0.01, vs. ACA group; ^b^P<0.01, vs. previous time point (n=6). U or UTI, ulinastatin; TNF, tumor necrosis factor; ACA, asphyxial cardiac arrest.

**Figure 5:**
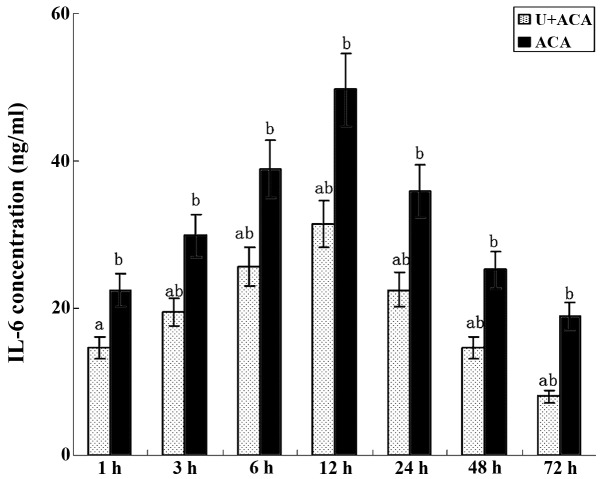
Effect of UTI on IL-6 levels. ^a^P<0.01, vs. ACA group; ^b^P<0.01, vs. previous time point (n=6). UTI, ulinastatin; IL, interleukin; ACA, asphyxial cardiac arrest.

As shown in Figs. 4 and 5, the ELISA results revealed changes in the cytokine levels similar to those observed for TLR4 gene expression and NF-κB activity. TNF-α and IL-6 were readily secreted in the brain cells at 1 h post-resuscitation. TNF-α levels peaked at 3 h and remained high prior to a significant decrease observed at 24 h (Fig. 4). IL-6 levels reached the highest value at 12 h and gradually decreased thereafter (Fig. 5). Minimal production of TNF-α and IL-6 was observed at 72 h post-resuscitation. Notably, significantly lower levels of TNF-α and IL-6 were observed in the UTI-treated animals at each time point when compared with the control group (P<0.05).

## Discussion

Patients that are successfully resuscitated from CA often experience neurological complications, including cognitive impairment and motor deficit, which potentially incapacitate the individuals and may result in mortality (2,3). Thus, providing an effective regimen for neurological injury attenuation following CPR is important. The TLR4 inflammatory signaling pathway has previously been reported to play a critical role in brain injury following CPR, since brain injury has been shown to be alleviated by inhibiting the TLR4 signaling pathway via hypoxic preconditioning (7). Therefore, hypoxia-based strategies have been widely used to investigate the mechanisms involved in the pathogenesis of cerebral ischemic injury. However, since the onset of CA is unpredictable, hypoxic preconditioning is not applicable for patients experiencing CA. UTI, a urinary trypsin inhibitor, has been shown to possess anti-inflammatory properties and attenuate LPS-induced acute lung injury (10), inhibit systemic inflammatory responses resulting from pulmonary I/R (11) and alleviate pulmonary I/R injury via the inhibition of TNF-α expression in rats (17). In addition, previous studies have demonstrated that UTI suppresses the elevation of IL-6 and IL-8 levels in patients undergoing coronary artery bypass grafting under extracorporeal circulation (18), alleviates forebrain I/R injury by inhibiting the production of superoxide radicals and intercellular adhesion molecule-1 (19) and improves oleic acid-induced acute lung injury by reducing TNF-α levels and inducing leukocyte activation (20). In addition, UTI has been proposed as a therapeutic option for endotoxin-associated inflammatory disorders, including acute lung and liver injuries (21), due to its anti-inflammatory properties (22–24). However, whether UTI protects against brain injury by inhibiting the inflammatory responses following CA is yet to be elucidated. Therefore, a prospective rat study was conducted to evaluate the role of UTI. Compared with the control group, the mortality rate was significantly reduced following UTI treatment at 24 h after resuscitation from CA. In addition, the NDS scores were markedly improved in the treatment group when compared with the control animals at 24, 48 and 72 h post-resuscitation.

TLR4 is a pattern recognition receptor that predominantly recognizes endotoxins. A previous study demonstrated that TLR4 can bind to various exogenous ligands, including heat shock proteins, protein fragments from the extracellular matrix, hyaluronan, heparitin sulfate, vascular fibrin monomer-fibrinogen complex and phylaxin and elastase released from immune cells (25). The suppression of exogenous substance release by UTI may contribute to the inhibitory effects on TLR4 expression. However, further studies are required to clarify these possible mechanisms.

Once TLR4 is activated, the interaction of the TLR4 intracellular domains with the adapter protein, MyD88, promotes the expression of IL-1 receptor-associated kinase and TNF receptor-associated factor 6, which then upregulate NF-κB. Activated NF-κB further stimulates the expression of various inflammatory cytokines, including TNF-α, IL-1 and IL-6, resulting in inflammation (26). TNF-α and IL-6 are the most important cytokines regulated by NF-κB and are considered to play a central role in the development of inflammatory diseases. TNF-α levels have been shown to positively correlate with mortality rates, with high levels inducing hypotension, tissue injury and consequently mortality in animals (27). In addition, IL-6 plasma levels have been demonstrated to be closely associated with survival and mortality (28). Based on these observations, the present study was designed to detect the changes in the levels of NF-κB, TNF-α and IL-6 in a rat model of CPR following asphyxial CA. Significant elevations in the levels of TLR4 gene expression and NF-κB activity were observed, as well as markedly increased levels of TNF-α and IL-6 in the brains tissues of rats resuscitated from CA at each time point. These observations indicate that UTI treatment at the onset of CPR significantly suppresses the TLR4/NF-κB signaling cascade following resuscitation, thereby alleviating the inflammatory responses in the brain. The overt reduction in the mortality rate and the improvement in the NDS scores were likely to have resulted from UTI exerting anti-inflammatory properties in the brain tissues.

In conclusion, UTI was found to alleviate brain injury following CPR by inhibiting the TLR4 signaling pathway and reducing the release of inflammatory cytokines; thus, exerting anti-inflammatory effects.

## Figures and Tables

**Table I:** Modified NDS scores.

Parameter	Points	Maximum points
Arousal		19
Alerting (normal/stuporous/comatose)	10/5/0	
Eye opening (open spontaneously/open to pain/absent)	3/1/0	
Spontaneous respiration (normal/abnormal/absent)	6/0/0	
Brainstem function		21
Olfaction (present/weak/absent)	3/1/0	
Vision (present/weak/absent)	3/1/0	
Papillary light reflex (present/weak/absent)	3/1/0	
Corneal reflex (present/weak/absent)	3/1/0	
Startle reflex (present/weak/absent)	3/1/0	
Whisker stimulation (present/weak/absent)	3/1/0	
Swallowing (present/weak/absent)	3/1/0	
Motor assessment (each side tested and scored separately)		6
Strength (normal/weak movement/no movement)	3/1/0	
Sensory assessment (each side tested and scored separately)		6
Pain (brisk withdrawal/weak withdrawal/no movement)	3/1/0	
Motor behavior		6
Gait coordination (normal/abnormal/absent)	3/1/0	
Balance during walking (normal/abnormal/absent)	3/1/0	
Behavior		12
Righting reflex (normal/abnormal/absent)	3/1/0	
Negative geotaxis (normal/abnormal/absent)	3/1/0	
Visual placing (normal/abnormal/absent)	3/1/0	
Turning alley (normal/abnormal/absent)	3/1/0	
Seizures (no seizure/focal seizure/generalized seizure)	10/5/0	10
Feeding (normal/abnormal/absent)	10/5/0	10
Grooming (normal/abnormal/absent)	10/5/0	10

100, normal; 0, brain dead; NDS, neurological deficit scale.

**Table II:** Comparison of mortality rates, TCA, TROSC, BWC and NDS scores between the control and treatment groups.

Variable	Treatment group	Control group
Mortality rate, (n=50)	2/50 (4)^a^	6/50 (12)
TCA, sec (n=50)	190.3±19.6	191.1±20.8
TROSC, sec (n=42)	73.9±22.4^a^	99.3±16.6
BWC (n=6)	77.33±4.5 a	84.06±1.83
NDS (n=6)		
24 h	69.44±6.3^a^	54.38±6.8
48 h	73.77±5.69^a^,^b^	63.44±6.48^b^
72 h	88.55±5.66^a^,^c^	70.44±8.62^c^

Results are expressed as the mean ± SD. BWC = (wet weight − dry weight)/wet weight × 100.

a P<0.01, vs. control group;

b P<0.05, vs. 24 h post-resuscitation;

c P<0.05, vs. 48 h post-resuscitation;

TCA, time to cardiac arrest; TROSC, time to return of spontaneous circulation; BWC, brain water content; NDS, neurological deficit score; SD, standard deviation.
